# Swept-source optical coherence tomography and optical coherence tomography angiography in acquired toxoplasmic chorioretinitis: a case report

**DOI:** 10.1186/s13256-018-1902-x

**Published:** 2018-12-04

**Authors:** Diego Vezzola, Davide Allegrini, Alfredo Borgia, Paolo Fogagnolo, Luca Mario Rossetti, Mario R. Romano, Stefano De Cillà

**Affiliations:** 1 Eye Unit, University Hospital Maggiore della Carità, Novara, Italy; 2 Eye Clinic, Humanitas Gavazzeni Hospital, Humanitas University, Bergamo, Italy; 3 Eye Clinic, San Paolo Hospital, University of Milan, Milan, Italy

**Keywords:** Angio-OCT, Swept-source OCT, Toxoplasmic uveitis

## Abstract

**Purpose:**

To describe swept-source optical coherence tomography and optical coherence tomography angiography retinal changes in a case of acute toxoplasmic chorioretinitis both at the time of diagnosis and after healing.

**Case presentation:**

A 57-year-old white woman suffering from acquired toxoplasmic chorioretinitis underwent swept-source optical coherence tomography and optical coherence tomography angiography both at the time of diagnosis and after healing. In the acute phase of the disease, swept-source optical coherence tomography clearly showed retinal and choroidal involvement in the superficial retina and in the choroidal swelling. Optical coherence tomography angiography showed a complete loss of deep and superficial capillary networks and of choroidal vessels in the area of the inflammation. After healing, swept-source optical coherence tomography showed a retinal thinning of the area involved, with a subversion of retinal layers and no visible change at the choroid level. On the other hand, optical coherence tomography angiography showed the persistence of a vascular occlusion at the retina and choroid level.

**Conclusion:**

This is the first case in the optical coherence tomography angiography literature that shows the imaging of toxoplasmic chorioretinal lesions. This case confirms the involvement of the retina and choroid in toxoplasmic uveitis and the disruptive potential of such inflammation. The optical coherence tomography angiography performed after healing showed a persistent ablation of the retina, choriocapillaris, and choroidal vessels. The non-invasive optical coherence tomography angiography imaging technique may have diagnostic and prognostic value in regard to toxoplasmic uveitis.

## Introduction

*Toxoplasma gondii* is a ubiquitous intracellular parasite deemed to be the leading infectious cause of posterior uveitis worldwide [1]. In high *T. gondii* endemic regions of the USA and Europe, ocular toxoplasmosis is the most frequent cause of posterior uveitis [2, 3]. Recent reports indicated that acquired infections affect a larger ocular portion than congenital ocular toxoplasmosis [4, 5].

Ocular toxoplasmosis is commonly detected by means of standard ophthalmic researches and its diagnosis is obtained through a clinical examination [6]. Seropositivity to *T. gondii* infection indicates a previous systemic exposure to the parasite. Toxoplasmic chorioretinitis can be congenital or acquired. The congenital form is due to a systemic maternal infection during pregnancy and causes scars in the fetus retina, which are often bilateral with macular involvement [7]. The acquired form appears in most cases (72%) as recurrence of a previous asymptomatic infection, with a concentration of necrotizing retinitis or retinochoroiditis adjacent to a variably pigmented chorioretinal scar. Usually, recurrences affect younger patients (mean age 30 years) with a positive immunoglobulin (Ig) G serology. However, older patients (mean age 51 years), in most cases, experience an acute primary chorioretinal lesion during a primary systemic infection, depending on their serology [7]. Active lesions are associated with symptomatic vitreous inflammation, which is the presenting symptom of ocular toxoplasmosis, which leads to blurred vision. The classical ocular manifestation of toxoplasmosis is a focal necrotizing retinitis or retinochoroiditis. A severe vitritis causes the classic “headlight in the fog” sign [8].

Swept-source optical coherence tomography (SS-OCT) is the latest milestone in retinal and choroidal imaging. Because its wavelength of 1050 nm, which is superior to the 840 nm of spectral domain optical coherence tomography (SD-OCT), it is able to overcome ocular opacities such as cataracts and vitritis, allowing the retinal and choroidal visualization of eyes whose fundus is not clearly visible.

For the same reason, SS-OCT allows visualization of the retinal and choroidal vascular networks, even in eyes with medium opacity [9]. Optical coherence tomography angiography (OCTA) is a non-invasive imaging technique that employs motion contrast imaging to high-resolution volumetric blood flow information generating angiographic images. Optical coherence tomography (OCT) angiograms are *en face* images which can be scrolled outward from the internal limiting membrane (ILM) up to the choroid in order to visualize the individual vascular plexus and segment the inner retina, outer retina, choriocapillaris, or other areas of interest [10]. In this case report we describe the SS-OCT and OCTA imaging techniques used in the case of a 57-year-old white woman suffering from primary acute toxoplasmic chorioretinitis. To the best of our knowledge, such a report has never been done before.

## Case presentation

**Fig. 1:**
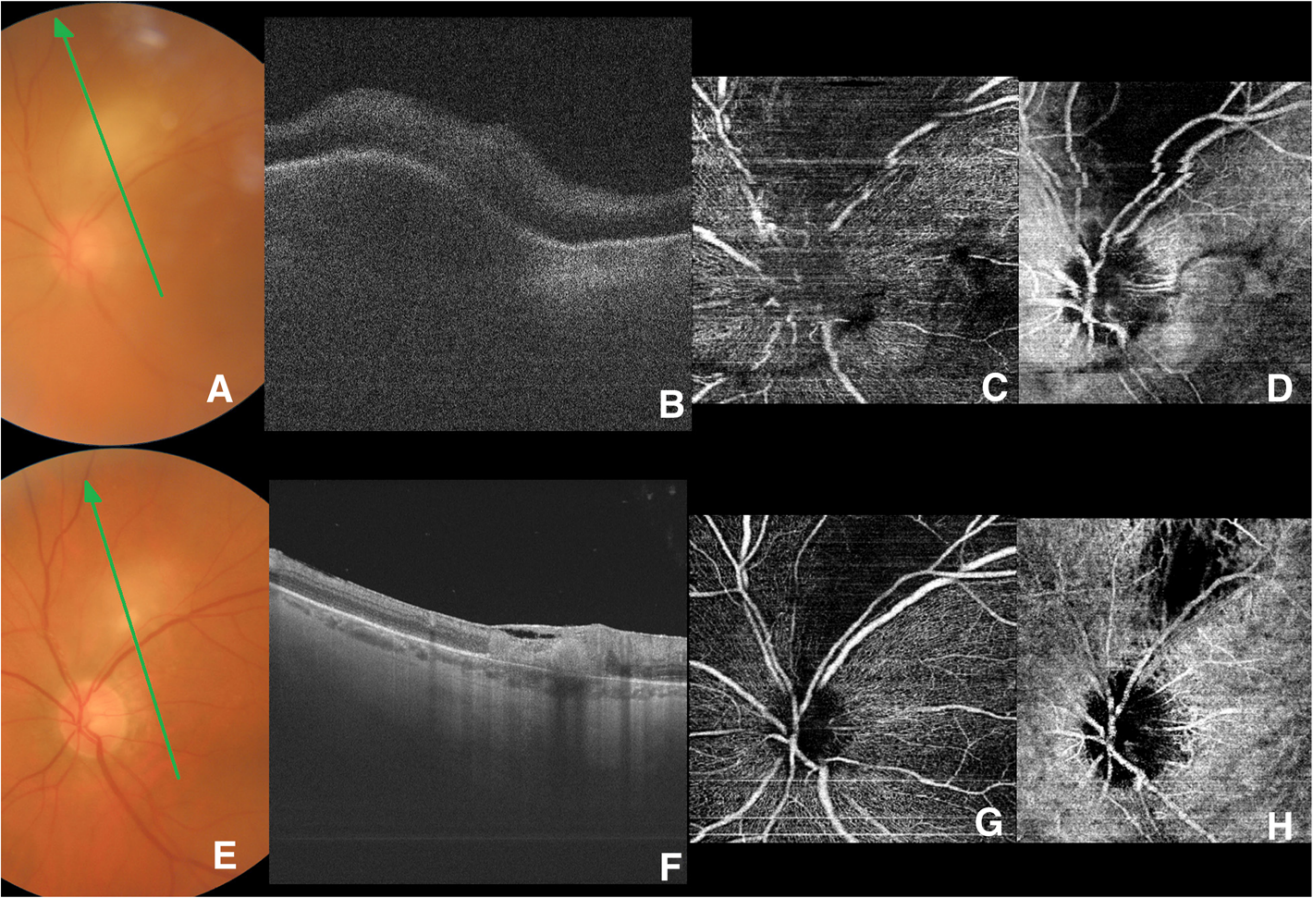
**a** Color fundus at time of diagnosis. Active toxoplasmic lesion is visible over the optic disc despite blurring by vitreitis. Previous pigmented lesions are absent. **b** A 6 mm swept-source optical coherence tomography crossing the lesion area (see *green arrow* in **a** for scan direction): despite blurring, vitreitis nerve fiber swelling and choroidal thickening are well visible. **c** Superficial retina and (outer retinal layer). **d** Optical coherence tomography angiography over the lesion shows superficial retinal plexus (**c**) and choriocapillaris and outer retinal layer (**d**). Vitreous shading due to inflammatory opacities is clearly visible under the optic disc. **e** Color fundus 2 months after therapy: inflammatory signs are absent but an atrophic scar is visible. **f** A 6 mm swept-source optical coherence tomography crossing the lesion (see *green arrow* in **e** for scan direction): retinal layers are no longer recognizable at the site of the lesion. Epiretinal membrane appeared possibly due to inflammatory reaction. Choroid has a normal thickness. **g** and **h** Optical coherence tomography angiography over the lesion shows only partial retina capillary plexus (**g**) and deep choroidal vessels (**h**) reperfusion. In **h** only deep choroidal vessels are evident in the affected area with persistent choriocapillaris failure

A 57-year-old white woman presented to the emergency department of the hospital in Novara, Italy, with sudden ocular pain and blurred vision in her left eye. The best corrected visual acuity (BCVA) was 20/20 in her right eye and 20/200 in her left eye. Her right eye was normal. Her left eye anterior segment showed an aqueous flare and the presence of cells and keratic precipitates without posterior synechiae. Intraocular pressure was 14 mmHg in her right eye and 20 mmHg in her left eye. A fundus examination showed an intense vitreitis and a focal necrotizing retinochoroiditis above the optic disc (Fig. 1a). As the pigmented chorioretinal scar adjacent to the active lesion was not visible, a clinical diagnosis of suspected primary ocular toxoplasmosis was made. The diagnosis had been confirmed by anamnestic data (our patient adopted a wildcat a few months before). Serology for *T. gondii* (IgM and IgG) was positive, but it was negative for other common uveal infections. Necrotic herpetic retinopathies, cytomegalovirus retinitis, syphilitic chorioretinitis, and tuberculous chorioretinitis were excluded by serological tests. In addition, a chest X-ray to detect hilar adenopathy of sarcoidosis was performed. The result was negative. After that, a systemic antibiotics combination therapy with pyrimethamine and sulfadiazine was performed and a steroid therapy with prednisone was started. Two months after healing, the inflammation completely disappeared. Analyzing the fundus, a non-pigmented atrophic chorioretinal scar without signs of vitreitis was clearly visible in the primary lesion site and a clinical examination showed that the BCVA was 20/25 in her left eye.

Despite the vitreitis (Fig. 1b–d, f–h), SS-OCT and OCTA (Triton Plus®, Topcon Corporation, Tokyo, Japan) were performed before and after the resolution of the disease. In particular, the SS-OCT showed nerve fiber swelling and choroidal thickening (Fig. 1b). The OCTA performed on the lesion showed an obliteration of the retinal capillary, choriocapillary, and deep choroidal vessels (Fig. 1c, d). Fluorescein angiography (FA) showed a hypofluorescence during the early phase, followed by a progressive hyperfluorescence that developed after the leakage phase; on the other hand, indocyanine green angiography (ICGA) showed a hypofluorescent lesion with characteristic hypofluorescent perilesional satellite lesions.

After healing, an SS-OCT performed on the lesion showed a hyper-reflective dome-shaped intraretinal mass involving the entire retinal thickness, associated with an increasing retinal thickness on the scar site (Fig. 1f). The retinal layers within the mass could no longer be recognized. The choroid had a normal thickness. An OCTA performed on the lesion showed a persistent retinal capillary plexus and a choriocapillaris failure with partial reperfusion of the deep choroidal vessels (Fig. 1g, h).

## Discussion

Non-invasive clinical imaging with SS-OCT and OCTA confirms that toxoplasmic uveitis affects the vitreous and all the retinal layers up to the choroid, causing inflammatory swelling of the retina and choroid. As previously described by Chen *et al.*, despite the presence of intense vitreitis [11], the use of 1050 nm wavelength SS-OCT can provide better images than SD-OCT and TM-OCT. It also allows us to verify the involvement of the vitreous, retina, and choroid with a single scan image.

In this study we report for the first time OCTA images of toxoplasmic chorioretinitis from an acute stage to a quiescent stage. The OCTA confirmed the results of previous FA and ICGA studies [12] by showing a vascular obliteration of the retina and choroid, which is typical of toxoplasmic inflammatory lesions. After the resolution of the disease, the vascular obliteration appears to be only partially reversible in the periphery of the lesion, although not in the center. Furthermore, the choriocapillaris texture destruction persists around the obliterated area and only the deep choroidal vessels remain visible.

By performing FA, the chorioretinal vascular network was not clearly visible because it was masked by a rapid loss of dye from the vessels. Instead, the OCTA allowed us to visualize the entire lesion more clearly. In fact, because of the absence of dye, there was no interference due to early leakage. Compared to FA, OCTA has the advantage of determining more accurately which retinal vascular layer is affected.

## Conclusion

The non-invasive OCTA and SS-OCT imaging techniques may help a physician to make the correct diagnosis, especially when a pigmented retinal scar is not visible. However, further studies in this regard are needed. Furthermore, OCTA may play a special role as a prognostic tool in regard to toxoplasmic uveitis. In particular, when the toxoplasmic lesion is close to the macula, OCTA may show the macula vascular involvement more effectively than FA. OCTA is also able to detect which retinal vascular layer is affected. In the current situation, in which the vascular obliteration in such pathology seems to be only partially reversible, OCTA may add valuable prognostic information for sight recovery after healing.

## Abbreviations

BCVA: Best corrected visual acuity
FA: Fluorescein angiography
ICGA: Indocyanine green angiography
Ig: Immunoglobulin
ILM: Internal limiting membrane
OCT: Optical coherence tomography
OCTA: Optical coherence tomography angiography
SD-OCT: Spectral domain optical coherence tomography
SS-OCT: Swept-source optical coherence tomography
